# Long-term effect of discontinuing anticholinesterase treatment on cognitive decline and mortality in Alzheimer's disease in France: a quasi-experiment and target trial emulation study

**DOI:** 10.1016/j.lanepe.2026.101607

**Published:** 2026-02-12

**Authors:** Simon Lecerf, Octave Guinebretiere, Raphaël Bentegeac, Victoria Gauthier, Estelle Aymes, Chaymae Mekkaoui, Julien Dumurgier, Philippe Amouyel, Stanley Durrleman, Thomas Nedelec, Thibaud Lebouvier

**Affiliations:** a Univ. Lille, Inserm, CHU Lille, Lille Neuroscience & Cognition, LabEx DISTALZ, F-59000, Lille, France; b Paris Brain Institute, ICM, Inserm U 1127, CNRS UMR 7225, Sorbonne Université, Inria, Aramis project team, F-75013, Paris, France; c Sorbonne Université, INSERM, Institut Pierre Louis d’Epidémiologie et de Santé Publique, Equipe PEPITES, AP-HP, Hôpital Pitié Salpêtrière, Département de Santé Publique, Centre de Pharmacoépidémiologie (Cephepi), Unité de Recherche Clinique PSL-CFX, F75013, Paris, France; d Univ. Lille, Inserm, Centre Hosp. Univ Lille, Institut Pasteur de Lille, UMR1167 – RID-AGE LabEx DISTALZ - Risk factors and molecular determinants of aging-related diseases, Epidemiology and Public Health Department, F-59000, Lille, France; e Université Paris-Cité, Cognitive Neurology Center, GHU APHP Nord Lariboisière Fernand-Widal Hospital, Paris, France

**Keywords:** Alzheimer's disease, Cholinesterase inhibitors, Cognitive decline, Emulation study, Health policy, Real-world data

## Abstract

**Background:**

In 2018, France withdrew reimbursement for cholinesterase inhibitors (ChEIs) in Alzheimer's disease, citing modest efficacy, lack of long-term benefit, and safety concerns. This policy shift provided a unique opportunity to assess ChEI effectiveness in real-world settings, by evaluating, among patients treated with ChEI between 01/08/2017 and 01/08/2018, the impact of treatment discontinuation on cognitive decline (MMSE score) and survival.

**Methods:**

Using the French National Alzheimer's Database (BNA) and Meotis databases, we emulated a pragmatic, intention-to-treat trial comparing patients who discontinued ChEIs after delisting to those who continued treatment, under quasi-experimental conditions. To model cognitive trajectories, we used the inverse probability treatment-weighted (IPTW) cohort and applied mixed-effects models with random intercepts across all follow-up visits. Survival was estimated with a pooled logistic regression including treatment group, follow-up time, and an interaction between treatment and time.

**Findings:**

The mean difference in MMSE decline between discontinuers and continuers after one year was 0·97 points (95% CI 0·68–1·27, *p* < 0·001), reaching 1·81 points (0·91–2·71, *p* < 0·001), after four years. No significant difference in mortality (RR 1·10, 95% CI [0·95–1·29]) was observed over a five-year period.

**Interpretation:**

Our findings confirm and extend prior trials by demonstrating the sustained cognitive benefits of ChEIs in a real-world setting. While acknowledging the limitations associated with its retrospective nature, our study argues for reconsidering the 2018 delisting decision, as ChEIs remain safe and clinically relevant for mild-to-moderate Alzheimer's disease.

**Funding:**

This research received no specific grant from any funding agency in the public, commercial, or not-for-profit sectors.

**Research in context**

**Evidence before this study**

We searched PubMed with the terms “donepezil”, “rivastigmine”, “galantamine”, “cholinesterase inhibitors”, and “Alzheimer's disease” for articles published in English or French up to December 2025. Randomised controlled trials of cholinesterase inhibitors mainly reported short-term cognitive benefits of 6 months, with few studies extending to 12 months. Long-term randomised trials were lacking or limited by high attrition and methodological complexity. Evidence from observational cohorts suggested potential long-term benefits, but these studies were prone to indication and selection bias.

**Added value of this study**

Our study leveraged the unique policy-driven withdrawal of reimbursement for cholinesterase inhibitors in France to emulate a “stop trial” under real-world conditions. Using two large databases, we compared cognitive trajectories and mortality between continuers and discontinuers. Our findings of a consistent, detrimental effect of discontinuation on cognition, equivalent to a 6- to 12-month delay in decline, recapitulate and extend those of earlier trials in a more representative population.

**Implications of all the available evidence**

Taken together, the evidence indicates that cholinesterase inhibitors provide modest but sustained symptomatic benefits in Alzheimer's disease, particularly when treatment is continued. Despite concerns regarding adverse effects, discontinuation appears to accelerate cognitive decline without a survival benefit. These findings support reconsidering France's reimbursement policy and reinforce the relevance of symptomatic treatments in the era of disease-modifying therapies.

## Introduction

In 2016, the French Health Authority's (HAS) Commission of Transparency ruled against reimbursing the cholinesterase inhibitors (ChEIs) donepezil, rivastigmine, and galantamine for the treatment of Alzheimer's disease (AD) for the following reasons: (1) efficacy in trials was modest; (2) long-term benefit was undemonstrated; and (3) generalisability to older patients and those with comorbidities was questioned, with potentially serious complication.^1^ Following this, the French Ministry of Health announced on 29th May 2018 the delisting of all ChEIs from 1st August 2018.^2^

While some specialists and general practitioners immediately complied, many were sceptical. The French Federation of Memory Centres and patient associations lodged an unsuccessful appeal with the Conseil d'État.^3^ As the European market authorisation remained valid, ChEIs stayed available in pharmacies at €15–35 per month, their prescription remaining limited to memory centre specialists. In this context, patients mostly stopped or continued treatment based on their doctor's advice.^4^ An observational study using the Banque Nationale Alzheimer (BNA) database found that 19·5% of patients discontinued treatment after reimbursement ended, compared to 7·5% the previous year.^5^ No significant link with socioeconomic factors was observed. Another study reported a 48% decrease in AD drug sales in summer 2018.^6^

ChEIs increase acetylcholine in the brain, a neurotransmitter associated with attention and memory, by inhibiting its hydrolysing enzyme. In most developed countries, ChEIs are first-line therapy for mild-to-moderate AD.^7^ However, since the marketing of donepezil in 1996, long-term efficacy and optimal stopping time have been questioned due to a lack of long-term randomised clinical trials (RCTs). Although favourable answers have been obtained from an extensive retrospective study comparing ChEI-treated patients with a propensity score–matched cohort,^8^ these results may still be influenced by selection bias favouring healthier patients.

Another way to assess long-term efficacy is a stop trial. In this regard, the multicentre, double-blind, placebo-controlled DOMINO trial, which examined maintaining or stopping donepezil in moderate-to-severe AD, serves as a model.^9^ The 2018 French delisting presents a unique opportunity for a stop-trial emulation in mild-to-moderate AD. Real-world trial emulation reduced external validity bias, and the decision to continue or discontinue treatment was less medically driven in this context, thereby reducing selection bias. Using the BNA and Meotis databases, we thus emulated an intention-to-treat design of a pragmatic randomised trial based on DOMINO methodology.^9^

## Methods

### Design

We explicitly emulated a target trial to assess the effect of discontinuing ChEIs on cognition and mortality (Table 1), using data from the BNA and Meotis specialised memory centres. The trial design was inspired by the DOMINO study,^10^ and our protocol closely followed its components wherever possible. Some adaptations were necessary: (1) Mini-Mental State Examination (MMSE) score^11^ eligibility criteria were modified to reflect French prescribing guidelines, and (2) additional methodological adjustments were made to accommodate observational data. Treatment group assignment was based on patients’ baseline medication records. We assumed that the two groups were exchangeable at baseline, conditional on the covariates, and that the quasi-experimental context of the ChEI delisting in France mitigated potential unmeasured confounding.

**Table 1:** Specification and emulation of a target trial of ChEI and cognition trajectory using BNA and Meotis observational data.

Protocol component	Target trial specification	Target trial emulation
Eligibility criteria	• Age >18, between Aug 1 2017 and Aug 1 2018. • MMSE >10. • Diagnosed with AD. • Treated with ChEI during the past six months. • Baseline is defined as the first month in which all eligibility criteria are met.	Same as for target trial
Treatment strategies	• Discontinuation of ChEI at baseline. • Continuation of ChEI at baseline.	Same as for target trial
Treatment assignment	Individuals are randomly assigned to a strategy at baseline and will be aware of the strategy to which they have been assigned	We classified individuals according to the strategy that their data were compatible with and attempted to emulate randomisation by adjusting for baseline confounders (age, sex, MMSE, psychoanaleptics, cardiovascular treatments, memory centre, housing, education)
Follow-up	For each eligible individual, follow-up begins at assignment (baseline or time zero) and continues until death, loss to follow-up, or administrative end of follow-up, whichever occurred first.	Same as for the target trial
Outcome	• MMSE score trajectories' difference. • MMSE score difference at one, two, three, and four years form baseline. Mortality.	Same as for target trial
Causal contrast	Intention to treat	Observational analogue of the intention-to-treat
Data analysis	Intention-to-treat analysis	Same as for the target trial ITT analysis except we emulated a sequence of target trials beginning on each month during the period from Aug 1 2017 to Jul 31, 2018. We used IP weighting to adjust for baseline confounding.

ITT = intention-to-treat, IP = inverse probability, ChEI = (Cholinesterase inhibitor), AD = Alzheimer's disease, MMSE = Mini Mental State Examination.

### Data sources and approvals

We used the BNA and Meotis databases. The BNA, established in 2010, contains systematically collected data from all patients attending memory centres in mainland France.^12^ Some data tend to be exhaustive, as they serve to measure centre activity and determine funding. The BNA is not designed as a study with specific inclusion or exclusion criteria. A unique anonymous key allows longitudinal follow-up of individual patients. Patient data include demographics, education level, and living situation, as well as diagnostic information at three levels: cognitive status (normal, mild, or major impairment), type of cognitive impairment, and aetiology (AD or related disorders). Consultation data include date of consultation, diagnosis, and MMSE score. Pharmacological (anti-dementia drugs and psychotropics) and non-pharmacological treatments are also recorded, though specific drug names and dosages are not included. The part of our project using the BNA database was approved by the Scientific Committee of the BNA (attestation BNA-CS-2025-03-008).

Founded in 1993, the Méotis network is the first French memory centre network, comprising 32 centres in the Nord and Pas-de-Calais departments, which have shared data in a common patient database since 1997. By 2025, the Meotis database included over 140,000 patients. All data are monitored and managed by a data manager at the Memory Resources and Research Centre (MRRC) of Lille University Hospital (MRRC/CMRR is the established name for university memory centres in France).^13^ Unlike the BNA, the date of death can be reliably retrieved from the Meotis database by consulting the French Public Data Platform (www.insee.fr). The part of our project using the Meotis database was conducted in accordance with French regulations for MR-004 type studies: the data processing was declared to the Data Protection Officer of Lille University Hospital (attestation DEC25-221), and all procedures were performed in compliance with applicable data protection regulations. Patients were informed of the study through appropriate means.

Data cleaning procedures were applied to ensure data quality. Implausible MMSE values that were inconsistent with subsequent assessments were excluded.

Due to differences in available outcome data, the BNA database was used for the cognitive trajectory study, and the Meotis database for the mortality study. More data on French dementia services can be found in this reference.^13^

### Eligibility criteria and baseline definition

Eligible individuals were over 18 years old, diagnosed with AD, had a baseline MMSE score of at least 10, and were registered in the BNA or Meotis database between 1st August 2017 and 1st August 2018. They also had to have received ChEI treatment in the past six months. Because treatment status was subject to interval censoring—patients were observed taking the medication at one visit and no longer taking it at the next—we used a conservative assumption that discontinuation occurred at the visit immediately preceding the first record of non-use, which likely underestimates the treatment effect. To reduce uncertainty in the inferred discontinuation date, we excluded individuals whose first follow-up visit occurred more than one year after baseline. Baseline was defined as the month when the eligibility criteria were met.

### Treatment assignment

Individuals were assigned to groups based on their baseline ChEI use: those not taking ChEI were assigned to the discontinuation group, and those still on treatment were assigned to the continuation group. We applied an intention-to-treat principle: patients assigned to the discontinuation group remained in that group throughout follow-up, even if they subsequently restarted ChEI treatment. Groups were assumed to be exchangeable at baseline, conditional on relevant covariates. For both cognitive trajectory and mortality, covariates included age, sex, MMSE, follow-up setting (memory centre or MMRC), living arrangement (home or nursing home), education level (primary, secondary, or post-secondary), and medications (antidepressants, neuroleptics, anxiolytics, hypnotics, and nootropics). For mortality, medication covariates additionally included antihypertensives, diabetes medications, and lipid-lowering agents.

### Outcomes and follow-up periods

We examined MMSE trajectories over the first four years after baseline. Unlike the DOMINO study, MMSE assessment times were not protocolised; we therefore estimated scores at any month using a mixed-effects model based on available patient visit data. Predicted MMSE scores were compared between groups at 1, 2, 3, and 4 years, and the delay in cognitive decline in the continuation group relative to the discontinuation group was evaluated at these same time points. The secondary outcome was all-cause mortality, compared between groups at five years. Individuals were followed from baseline until death, loss to follow-up, or the administrative end of follow-up (31st July 2024), whichever occurred first.

### Statistical analyses

Results are presented as mean (SD) unless stated otherwise.

We used the observational analogue of the intention-to-treat effect as a causal contrast: being assigned to ChEI discontinuation *versus* continuation at baseline.

We emulated the target trial as a series of trials starting at each of the 12 months between August 1, 2017, and August 1, 2018, such that each individual could participate in multiple trials (Supplementary Figures 1 and 2). This accommodates the fact that individuals may meet the eligibility criteria at several times over follow-up and is more statistically efficient than choosing just one of those times as baseline.^14^ We merged the data from the 12 emulated trials into a single dataset, treating the month of inclusion as a baseline covariate, and fitted mixed-effect and pooled logistic regression models to estimate the intention-to-treat effect on cognition and mortality, respectively. Variance of the estimates was calculated using bootstrapping to account for repeated inclusion of individuals.

Confounding by indication was accounted for using inverse probability of treatment weighting (IPTW). Each patient in every treatment group was assigned a weight equal to the inverse of their propensity score. Their propensity score was computed by estimating the probability of continuing treatment for continuers and of stopping treatment for discontinuers, based on all baseline covariates. Weights were stabilised by multiplying the inverse of the propensity score by the marginal probability of the treatment group. Weighting was deemed acceptable when the absolute standardised mean difference (SMD) among treatment groups was below 0·1.

### Cognitive trajectory

To model cognitive trajectories, we used the IPTW-weighted cohort and applied mixed-effects models with random intercepts across all follow-up visits. Individuals without any post-baseline MMSE assessment were also included in the mixed-effects model, without multiple imputation of their missing follow-up data, as it has been shown that multiple imputation is not necessary before performing a longitudinal mixed-model analysis.^15^ The model incorporated treatment group, follow-up time (quadratic spline with a knot at eight months, to account for potential symptomatic effects), and an interaction between time and treatment. MMSE trajectories were predicted from the model, and differences from baseline were computed. We calculated 95% confidence intervals (CIs) using non-parametric bootstrapping with 500 samples. Treatment differences in cognitive decline were derived by extracting predicted MMSE values for each group at each year, computing mean differences, and obtaining p-values using the percentile method or a bootstrap t-test, depending on data symmetry.

### Mortality

To build adjusted survival curves, we used the IPTW-weighted cohort to account for baseline confounders. The intention-to-treat effect was estimated using a pooled logistic regression model that included treatment group, follow-up time in months (flexible function with linear and quadratic terms), an interaction between treatment and time, and baseline month of the sequentially emulated trial. Absolute risks were estimated from model predictions, and 1- to 5-year risk ratios for death were computed comparing continuers to discontinuers. We calculated 95% confidence intervals (CIs) using nonparametric bootstrapping with 500 samples.

### Sensitivity analyses

As a sensitivity analysis and positive control, we replicated the DOMINO study, keeping all eligibility criteria except the baseline MMSE (set to 5–13 in DOMINO). In a second analysis, individuals were included only at their first eligibility time to check whether multiple inclusions or participation in both groups biased the treatment effect. A third analysis focused on individuals with prodromal/mild AD (baseline MMSE >20) to assess applicability to this subgroup. In a fourth analysis, we repeated the cognitive decline study in the Meotis database using the IPTW method, and then the inverse probability of censoring weighting (IPCW) method to account for potential bias from loss to follow-up. Finally, in a fifth sensitivity analysis, dropouts in the continuer group were assumed to be missing-not-at-random (MNAR). We conducted a pattern mixture model analysis using a delta-adjustment approach. Specifically, the predictive distribution of missing 1-year outcomes was obtained under the missing-at-random (MAR) assumption using a linear mixed-effects model, and predicted values for missing outcomes in the continuer group were shifted by a constant Δ value, while the discontinuer group remained unadjusted. The Δ value was incremented in steps of 0·5 to identify the Δ value at which the between-group difference in cognitive decline was no longer statistically significant at the two-sided 5% significance level.

### Role of the funding source

This research received no specific funding. The authors had full access to all the data and take responsibility for the integrity of the data and the accuracy of the data analysis.

## Results

### Participants

**Fig. 1:**
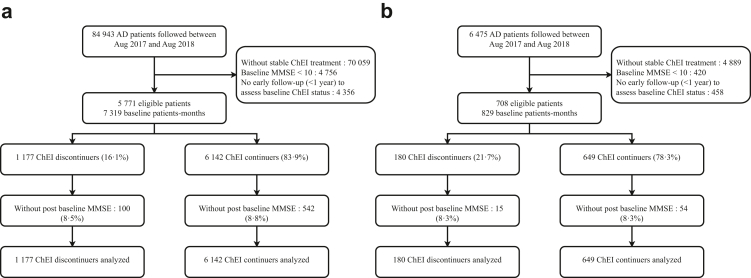
**Flowchart for selection of eligible patients for emulating two target trials of discontinuing ChEI effect on cognition and death.** (a) BNA database (cognition) (b) Meotis database (mortality). AD = Alzheimer's disease, ChEI = cholinesterase inhibitors, MMSE = Mini Mental State Examination.

Between 1st August 2017 and 1st August 2018, 5771 BNA and 708 Meotis patients met the inclusion criteria at least once (see flowchart, Fig. 1). This corresponded to 6142 BNA and 649 Meotis ChEI continuers, and 1177 and 180 discontinuers, respectively. The total exceeds the number of unique patients, as individuals could be included multiple times if they met eligibility during the enrolment period. Slightly over 8% (BNA: 642/7319; Meotis: 69/829) of patients had no post-baseline MMSE (but were included in the model) in both the BNA and Meotis databases. In the BNA database, 17·5% (14,884/84,943) of patients were on stable ChEI treatment at baseline, while 16·1% (1177/7319) had discontinued treatment. Trends in preceding and following years confirmed the impact of the delisting policy (Supplementary Figure 3).

Baseline characteristics are shown in Table 2. In the BNA database, discontinuers were older (81·8 ± 7·1 vs 80·3 ± 7·6 years, *p* < 0·001), had lower MMSE scores (17·9 ± 4·6 vs 19·1 ± 4·4, *p* < 0·001), and lower education (11·6% [136/1177] vs 15·5% [951/6142] post-secondary, *p* = 0·002). They were less often followed in MRRCs (19·6% [231/1177] vs 28·7% [1761/6142], *p* < 0·001) and more likely to live in nursing homes (9·2% [108/1177] vs 5·8% [354/6142], *p* < 0·001). Psychotropic use did not differ. Similar patterns were observed in Meotis. No differences were found for the use of antidiabetic, antihypertensive, or lipid-lowering agents. Post-baseline, continuers had more MMSE assessments (BNA: 3·7 ± 2·1 vs 3·1 ± 1·8 visits, *p* < 0·001; Meotis: 4·0 ± 2·4 vs 3·0 ± 1·5 visits, *p* < 0·001).

**Table 2:** Baseline characteristics of eligible individuals when emulating a target trial of the effect of discontinuation of ChEI on cognition (BNA) and mortality (Meotis), stratified by treatment status.

	BNA (cognition)			Meotis (mortality)		
	Discontinuers (N = 1177)	Continuers (N = 6142)	*p*-value	Discontinuers (N = 180)	Continuers (N = 649)	*p*-value
Demographics						
MMSE at baseline, mean (SD)	17·9 (4·61)	19·1 (4·45)	<0·001	17·4 (4·86)	18·9 (4·54)	<0·001
Age at baseline (years), mean (SD)	81·8 (7·10)	80·3 (7·58)	<0·001	80·0 (7·31)	77·2 (9·26)	<0·001
Women, n (%)	781 (66·4%)	3945 (64·2%)	0·17	134 (74·4%)	446 (68·7%)	0·16
Centre, n (%)			<0·001			<0·001
Memory and research centre	231 (19·6%)	1761 (28·7%)		22 (12·2%)	242 (37·3%)	
Memory centre	946 (80·4%)	4381 (71·3%)		158 (87·8%)	407 (62·7%)	
Education level, n (%)			0·002			0·047
Primary	605 (51·4%)	2938 (47·8%)		103 (57·2%)	311 (47·9%)	
Secondary	436 (37·0%)	2253 (36·7%)		65 (36·1%)	265 (40·8%)	
Post-secondary	136 (11·6%)	951 (15·5%)		12 (6·7%)	73 (11·2%)	
Living arrangement, n (%)			<0·001			0·015
Home	1069 (90·8%)	5788 (94·2%)		149 (84·2%)	517 (91·0%)	
Nursing home	108 (9·2%)	354 (5·8%)		28 (15·8%)	51 (9·0%)	
Medication at baseline, n (%)						
Antidepressants	445 (37·8%)	2227 (36·3%)	0·33	67 (37·2%)	277 (42·7%)	0·22
Neuroleptics	64 (5·4%)	277 (4·5%)	0·19	7 (3·9%)	33 (5·1%)	0·64
Anxiolytics	176 (15·0%)	870 (14·2%)	0·51	44 (24·4%)	137 (21·1%)	0·39
Hypnotics	65 (5·5%)	282 (4·6%)	0·19	23 (12·8%)	70 (10·8%)	0·19
Nootropics	9 (0·8%)	49 (0·8%)	1	2 (1·1%)	5 (0·8%)	0·046
Antihypertensive agents	N.I.	N.I.	N.I.	123 (68·3%)	410 (63·2%)	0·21
Antidiabetic agents	N.I.	N.I.	N.I.	29 (16·1%)	91 (14·0%)	0·56
Lipid-lowering agent	N.I.	N.I.	N.I.	86 (47·8%)	297 (45·8%)	0·69

N.I. = not measured.

### Cognitive decline

**Fig. 2:**
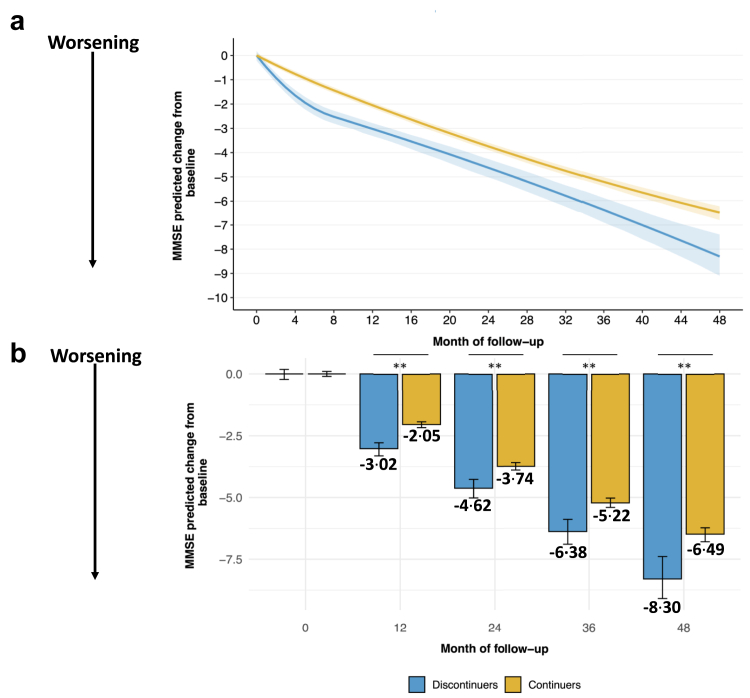
**Trajectories of cognition by treatment status predicted by the mixed effect model in the BNA database.** (a) Mean change from baseline in MMSE predicted from the mixed-effect model adjusted with inverse probability treatment weighting. (b) Comparison of mean change from baseline in MMSE score across treatments status at different time points. ∗∗p < 0·001.

Using the BNA database, baseline characteristics were well balanced after weighting (SMD ≤0·1, Supplementary Figure 4). ChEI continuers had a slower MMSE decline than discontinuers at one year (−2·05 95% CI [−2·17, −1·94] vs −3·02 [−3·32, −2·78], *p* < 0·001), two years (−3·74 [−3·89, −3·59] vs −4·62 [−5·02, −4·27], *p* < 0·001), three years (−5·22 [−5·41, −5·03] vs −6·38 [−6·90, −5·89], *p* < 0·001), and four years (−6·49 [−6·80, −6·23] vs −8·30 [−9·10, −7·39], *p* < 0·001) (Fig. 2). The corresponding differences were 0·97 [0·68, 1·27], 0·88 [0·48, 1·29], 1·16 [0·62, 1·70], and 1·81 [0·91, 2·71] MMSE points, reflecting a delay in cognitive decline of 6·5 [5·4, 7·5], 6·4 [4·1, 8·5], 7·7 [5·6, 9·9], and 11·3 [7·2, 14·5] months, respectively.

### Mortality

**Fig. 3:**
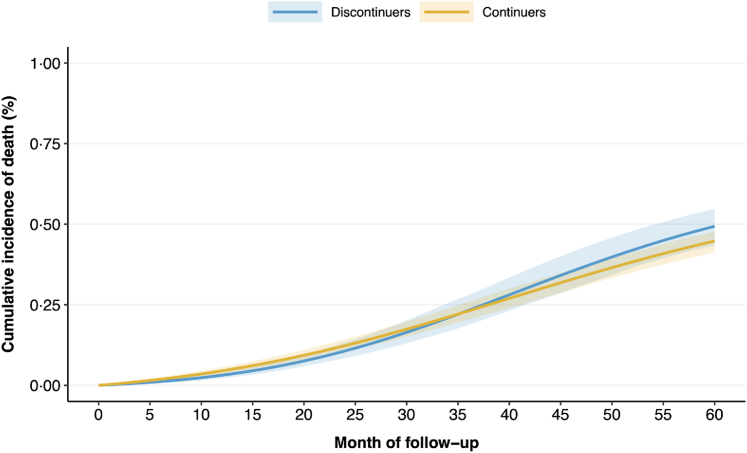
**Cumulative incidence of death curves in the discontinuer and continuer groups in the Meotis database.** We used the prediction from the pooled logistic regression and inverse probability method to obtain the survival.

Using the Meotis database, baseline characteristics were well balanced after weighting (SMD ≤0·1, Supplementary Figure 5). Cumulative incidence curves for death overlapped between treatment strategies (Fig. 3). Risk ratios for discontinuers versus continuers were not significant at one year (RR 0·66, 95% CI [0·41–1·05]), two years (0·84 [0·57–1·14]), three years (0·99 [0·80–1·26]), four years (1·08 [0·89–1·28]), and five years (1·10 [0·95–1·29]).

### Sensitivity analyses

Replicating the DOMINO study in the BNA database showed concordant results, with slower cognitive decline in continuers at one year (−1·98 [−2·25, −1·74] vs −3·33 [−3·71, −3·00], *p* < 0·001), a difference of 1·35 [0·92, 1·79] MMSE points (Supplementary Figures 6 and 7). A sensitivity analysis using only the first eligible point per individual yielded similar results, supporting the absence of bias and robustness of the primary analysis (Supplementary Figures 8–9). In patients with mild AD (MMSE >20), continuers also experienced a slower cognitive decline over one to four years: one year (−2·39 [−2·54, −2·26] vs −3·25 [−3·56, −2·89]), two years (−3·87 [−4·05, −3·69] vs −4·68 [−5·12, −4·19]), three years (−5·25 [−5·48, −5·02] vs −6·60 [−7·23, −5·92]), four years (−6·55 [−6·86, −6·24] vs −9·02 [−10·08, −7·94]), all *p* < 0·001 (Supplementary Figure 10), corresponding to differences of 0·86 [0·50, 1·22], 0·81 [0·32, 1·31], 1·35 [0·65, 2·05], and 2·47 [1·35, 3·58] MMSE points at one, two, three, and four years.

In the Meotis database, continuers showed slower decline at one year (−2·37 [−2·69, −2·04] vs −3·32 [−3·96, −2·75], *p* < 0·01, Supplementary Figure 11), though differences at two to four years were not statistically significant. Similar results were obtained using the IPCW method (Supplementary Figures 12–13).

Finally, as missing MMSE values in the continuer group might reflect discontinuation of both treatment and follow-up at the memory centre due to a poor cognitive outcome, we re-estimated the linear mixed-effects model in the BNA database using penalised imputed values. This sensitivity analysis, using a pattern-mixture model with progressively shifted imputations, showed that a shift of 6·5 MMSE points in the imputed penalised values for the continuer group was required for the between-group difference at one year to become non-significant (Supplementary Table 2).

## Discussion

Our emulated trial, conducted under quasi-experimental conditions following the delisting of ChEI in France, shows a detrimental effect of ChEI discontinuation on cognition. While acknowledging the possibility of residual indication biases, we established that patients who stopped treatment declined by about one MMSE point more at one year than continuers, a gap that widened to 1·81 points at four years. No difference in mortality was observed over five years.

These findings support the benefits of ChEIs. Most trials are limited to short-term endpoints of around six months, at which point a Cochrane meta-analysis showed a 1·37-point MMSE benefit over placebo.^7^ Few RCTs extended to one year, confirming similar effects.^16^^,^^17^ However, longer-term RCTs are limited. The methodologically complex AD2000 study remains an exception: after completing a 12-week RCT, patients were rerandomized to donepezil or placebo, with double-blind treatment continuing as deemed appropriate.^18^ Over two years, MMSE scores were 0·8 points higher in the donepezil group, with no effect on institutionalisation or disability. The trial was notoriously underpowered for long-term outcomes due to high attrition; notably, it also included multiple wash-out periods, which may have reduced the apparent benefit of a treatment that is primarily symptomatic rather than disease-modifying. Nevertheless, AD2000 raised concerns about the long-term efficacy of ChEIs and influenced the French delisting decision.^1^

To address the lack of long-term RCT data, cohort comparisons with untreated historical AD populations suggested benefit.^19^^,^^20^ Yet, such observational studies are prone to bias. A recent Swedish real-world study compared the trajectories of patients treated with ChEI with those of a propensity-score–matched untreated cohort over 5 years.^8^ Despite a robust methodology, the MMSE benefit was modest, raising questions about its clinical relevance in routine care. Additionally, despite multiple propensity-score adjustments, retrospective studies remain susceptible to confounding factors.

Another approach to evaluate long-term efficacy are the ‘stop trials’, where stable patients on treatment are randomised to either continue or discontinue. In that regard, the seminal DOMINO trial tested donepezil continuation versus withdrawal in community-dwelling patients with moderate-to-severe AD.^9^ Continuing donepezil resulted in a 1·9-point higher MMSE at one year, exceeding the predetermined minimal clinically important difference of 1·4.^21^ However, some selection bias may limit generalisability, as healthier patients were likely enrolled. The study also focused on later disease stages, where functional outcomes may outweigh cognitive scores.

Our study is original in assessing the cognitive impact of long-term ChEI discontinuation using real-world data at a stage (MMSE ≥10) with a clear indication for treatment. We exploited the nationwide delisting of ChEIs in France as a ‘policy-driven natural experiment’, which shifted prescribing practices (Supplementary Figure 1) and likely reduced confounding factors that typically influence treatment decisions. This allowed us to estimate ChEI ‘effectiveness’ rather than ‘efficacy’, reflecting performance in the real-world population of patients with suspected AD on chronic treatment. Our findings replicate the results of the DOMINO trial in an earlier disease stage and a more representative population. Despite the earlier stage, the mean age was 80 years compared to 77 years in DOMINO.^9^ This generalises the trial findings to routine care. While residual confounding cannot be ruled out in observational studies, consistency with RCT results strengthens confidence in our conclusions.

Another key finding is the temporal pattern of decline after ChEI discontinuation. The MMSE difference appeared quickly within the first months and then plateaued, increasing slightly from 0·97 at one year to 1·81 at four years. This suggests a primarily symptomatic, rather than disease-modifying, effect that is nonetheless sustained over time. Importantly, long-term results exceed the minimal clinically important difference set for DOMINO and are close to that established with anchor-based methods in mild AD.^22^ They correspond to a delay in disease progression of 6·5–11·3 months, which is likely clinically meaningful, particularly at earlier disease stages,^23^ consistent with our sensitivity analysis (Supplementary Figure 10).

Beyond long-term efficacy, the French Health Authority (HAS) also raised concerns about ChEI safety.^1^ In RCTs, 29% of participants in the ChEI group discontinued due to adverse events, compared with 18% in the placebo group,^7^ with similar rates observed in observational studies.^24^ Most side effects, resulting from parasympathetic overstimulation, are usually transient and reversible, although some may require treatment withdrawal.^25^ More concerning are increased risks of syncope, bradycardia, pacemaker insertion, and hip fracture reported in population-based studies and meta-analyses,^26^^,^^27^ which fuelled concerns about higher mortality in real-world practice.^1^ Nevertheless, in our research, slowing cognitive decline was not offset by a decrease in mortality, either in the short term or over the 5 years. These findings accord with post-delisting data linking ChEIs to lower risks of myocardial infarction, stroke, and overall mortality,^28^^,^^29^ as also observed in the Swedish cohort.^8^ However, beyond cognition, the continuation of ChEIs did not yield an additional survival benefit, possibly due to limited power. Consistently, no stop-trial has yet shown improved survival with ongoing ChEI therapy.^30^

Our study has several limitations. First, we could not assess functional status, such as the preservation of activities of daily living, an outcome typically reported in AD trials. Second, our study relied on routine care, unmonitored databases—although the data we used (MMSE scores and ChEI status) are robust, and outliers were rare enough to dispense with database cleaning. Another weakness is that two separate databases were needed, as no single cohort provided both national coverage and an adequate sample size for cognitive modelling and mortality data linkage. In addition, coverage of the BNA database is incomplete, which may affect the generalisability of our findings, and some cases of AD are likely to be missed, particularly those managed solely in the community and not referred to specialist centres. However, exact figures on annual AD incidence in France are lacking. Third, as data were collected in routine practice, the number of MMSE assessments varied between patients, and missing data might not be random (MNAR). To address potential bias due to loss to follow-up, we applied inverse probability of censoring weighting, with consistent results both with and without this adjustment; however, in this analysis, deaths were treated like other dropouts, which is not entirely correct. An alternative approach would have been to use principal stratification, restricting the analysis to patients who survived throughout follow-up, though this also raises concerns by excluding those who died. Finally, this analysis does not exclude the possibility of bias due to MNAR data. Indeed, the interruption in follow-up could be associated with a poor patient outcome, leading to MNAR data that underestimate the true cognitive decline in both groups, although not necessarily biasing the treatment effect itself. Nevertheless, we conducted a sensitivity analysis under the more conservative assumption that MNAR data could differentially affect the two groups. In this analysis the continuers with missing data would need to decline by 6·5 points more than expected at one year (equivalent to an 8·5-point decline, roughly four times the 2·05-point decline observed in the primary model) to render the between-group difference non-significant (Supplementary Table 2), which suggests that any potential bias from MNAR data does not fully account for the observed difference.

While symptomatic treatments such as ChEIs are often overshadowed by the development of disease-modifying therapies for AD, they remain relevant due to accessibility, safety, and modest yet reproducible cognitive benefits in mild-to-moderate stages. In the context of the 2018 ChEI delisting in France, our findings indicate a clear negative cognitive impact of discontinuation, without any compensatory benefits in mortality or adverse effects. Although only some patients discontinued, the policy significantly impacted new prescriptions and follow-up (Supplementary Figure 1),^6^ raising concerns about a potential loss of opportunity for French patients with AD and their caregivers. The reimbursement policy should be reconsidered in light of this evidence.

## Contributors

Simon Lecerf: Writing—review and editing, Writing—original draft, Data curation, Formal analysis, Methodology; Octave Guinebretiere: Writing—review and editing, Writing—original draft, Data curation, Formal analysis, Methodology; Raphaël Bentegeac: Writing—review and editing, Data curation, Methodology; Victoria Gauthier: Writing—review and editing, Data curation; Estelle Aymes: Writing—review and editing, Methodology; Chaymae Mekkaoui: Writing—review and editing, Data curation; Julien Dumurgier: Writing—review and editing, Data curation; Philippe Amouyel: Writing—review and editing; Stanley Durrleman: Writing—review and editing; Thomas Nedelec: Writing—review and editing, Writing—original draft, Data curation, Formal analysis, Methodology, Conceptualisation, Supervision; Thibaud Lebouvier: Writing—review, and editing, Writing—original draft, Data curation, Formal analysis, Methodology, Conceptualisation, Supervision.

Simon Lecerf and Octave Guinebretiere had full access to all raw data, performed the statistical analyses, and verified the data for accuracy. Thibaud Lebouvier had final responsibility for the decision to submit the manuscript for publication.

## Data sharing statement

No additional data are available. The data used in this study were obtained from the BNA and Meotis databases and cannot be shared due to privacy regulations. The study protocol and statistical analysis code are available from the corresponding author upon reasonable request.

## Declaration of interests

Thibaud Lebouvier reports grants and contracts from Eli Lilly and Eisai, and consulting fees from Eli Lilly, with all payments made to his institution (no personal remuneration); and support for attending meetings from Eli Lilly (CTAD 2025), all independent of this work.

Stanley Durrleman reports grants from Agence Nationale de la Recherche paid to his institution (no personal renumeration). Stanley Durrleman also reports holding stocks in Qairnel SAS.

All other authors declare no competing interests.

## References

[1] Haute Autorité de Santé (HAS), Commission de la Transparence (2016). Rapport d’évaluation des médicaments indiqués dans le traitement symptomatique de la maladie d'Alzheimer. https://www.has-sante.fr/jcms/pprd_2974197/fr/evaluation-2016-des-medicaments-alzheimer-interet-medical-insuffisant.

[2] Ministère des solidarités et de la santé (2018). Arrêté du 29 mai 2018 portant radiation de spécialités pharmaceutiques de la liste mentionnée au premier alinéa de l’article L. 162-17 du Code de la sécurité sociale. https://www.legifrance.gouv.fr/eli/arrete/2018/5/29/SSAS1804466A/jo/texte.

[3] Krolak-Salmon P., Dubois B., Sellal F. (2018). France will no more reimburse available symptomatic drugs against Alzheimer's disease. J Alzheimers Dis JAD.

[4] Garnier-Crussard A., Krolak-Salmon P. (2022). Des traitements symptomatiques qui ne sont plus remboursés mais qui sont toujours utilisés dans la maladie d'Alzheimer : pourquoi ?. Presse Médicale Form.

[5] Herr M., Ankri J., Diard C., Hiance-Delahaye A. (2021). Removal of drugs for Alzheimer's disease from the list of reimbursable drugs in France: analysis of change in drug use, disease management and cognition using the national Alzheimer data bank (BNA). Drugs Aging.

[6] Ben Hassen C., Tahir R., Singh-Manoux A. (2022). Ten-year trends in sales of alzheimer disease drugs in France compared with sales in Germany, Spain, and the UK. JAMA Health Forum.

[7] Birks J.S. Cholinesterase inhibitors for Alzheimer's disease - birks, JS - 2006 | Cochrane Library. https://www.cochranelibrary.com/cdsr/doi/10.1002/14651858.CD005593/full.

[8] Xu H., Garcia-Ptacek S., Jönsson L., Wimo A., Nordström P., Eriksdotter M. (2021). Long-term effects of cholinesterase inhibitors on cognitive decline and mortality. Neurology.

[9] Howard R., McShane R., Lindesay J. (2012). Donepezil and memantine for moderate-to-severe Alzheimer's disease. N Engl J Med.

[10] Jones R., Sheehan B., Phillips P. (2009). DOMINO-AD protocol: donepezil and memantine in moderate to severe Alzheimer's disease - a multicentre RCT. Trials.

[11] Folstein M.F., Folstein S.E., McHugh P.R. (1975). ‘Mini-mental state’. A practical method for grading the cognitive state of patients for the clinician. J Psychiatr Res.

[12] Tifratene K., Robert P., Metelkina A., Pradier C., Dartigues J.F. (2015). Progression of mild cognitive impairment to dementia due to AD in clinical settings. Neurology.

[13] Chen Y., Lebouvier T., Skrobala E. (2020). Twenty-year trends in patient referrals throughout the creation and development of a regional memory clinic network. Alzheimers Dement N Y N.

[14] García-Albéniz X., Hsu J., Hernán M.A. (2017). The value of explicitly emulating a target trial when using real world evidence: an application to colorectal cancer screening. Eur J Epidemiol.

[15] Twisk J., de Boer M., de Vente W., Heymans M. (2013). Multiple imputation of missing values was not necessary before performing a longitudinal mixed-model analysis. J Clin Epidemiol.

[16] Winblad B., Engedal K., Soininen H. (2001). A 1-year, randomized, placebo-controlled study of donepezil in patients with mild to moderate AD. Neurology.

[17] Mohs R.C., Doody R.S., Morris J.C. (2001). A 1-year, placebo-controlled preservation of function survival study of donepezil in AD patients. Neurology.

[18] Courtney C., Farrell D., Gray R. (2004). Long-term donepezil treatment in 565 patients with Alzheimer's disease (AD2000): randomised double-blind trial. Lancet Lond Engl.

[19] Grossberg G., Irwin P., Satlin A., Mesenbrink P., Spiegel R. (2004). Rivastigmine in Alzheimer disease: efficacy over two years. Am J Geriatr Psychiatry.

[20] Small G.W., Kaufer D., Mendiondo M.S., Quarg P., Spiegel R. (2005). Cognitive performance in Alzheimer's disease patients receiving rivastigmine for up to 5 years. Int J Clin Pract.

[21] Howard R., Phillips P., Johnson T. (2011). Determining the minimum clinically important differences for outcomes in the DOMINO trial. Int J Geriatr Psychiatry.

[22] Muir R.T., Hill M.D., Black S.E., Smith E.E. (2024). Minimal clinically important difference in Alzheimer's disease: rapid review. Alzheimer's Dement.

[23] Petersen R.C., Aisen P.S., Andrews J.S. (2023). Expectations and clinical meaningfulness of randomized controlled trials. Alzheimers Dement J Alzheimers Assoc.

[24] Park K.H., Yang Y., Chen C. (2021). Discontinuation rate of newly prescribed donepezil in Alzheimer's disease patients in Asia. J Clin Neurol Seoul Korea.

[25] Herrmann N., Ismail Z., Collins R. (2022). CCCDTD5 recommendations on the deprescribing of cognitive enhancers in dementia. Alzheimers Dement N Y N.

[26] Gill S.S., Anderson G.M., Fischer H.D. (2009). Syncope and its consequences in patients with dementia receiving cholinesterase inhibitors: a population-based cohort study. Arch Intern Med.

[27] Kim D.H., Brown R.T., Ding E.L., Kiel D.P., Berry S.D. (2011). Dementia medications and risk of falls, syncope, and related adverse events: meta-analysis of randomized controlled trials. J Am Geriatr Soc.

[28] Isik A.T., Soysal P., Stubbs B. (2018). Cardiovascular outcomes of cholinesterase inhibitors in individuals with dementia: a meta-analysis and systematic review. J Am Geriatr Soc.

[29] Tan E.C.K., Johnell K., Garcia-Ptacek S. (2018). Acetylcholinesterase inhibitors and risk of stroke and death in people with dementia. Alzheimers Dement.

[30] Parsons C., Lim W.Y., Loy C. (2021). Withdrawal or continuation of cholinesterase inhibitors or memantine or both, in people with dementia. Cochrane Database Syst Rev.

