# Supplementary material for: Long-term effect of discontinuing anticholinesterase treatment on cognitive decline and mortality in Alzheimer's disease in France: a quasi-experiment and target trial emulation study
Source: Lancet Reg Health Eur. 2026 Feb 12;62:101607. doi: 10.1016/j.lanepe.2026.101607 (PMC12925193; doi:10.1016/j.lanepe.2026.101607)
Supplement: Supplementary Figures and Tables [file mmc1.docx]

**Supplementary data**

**
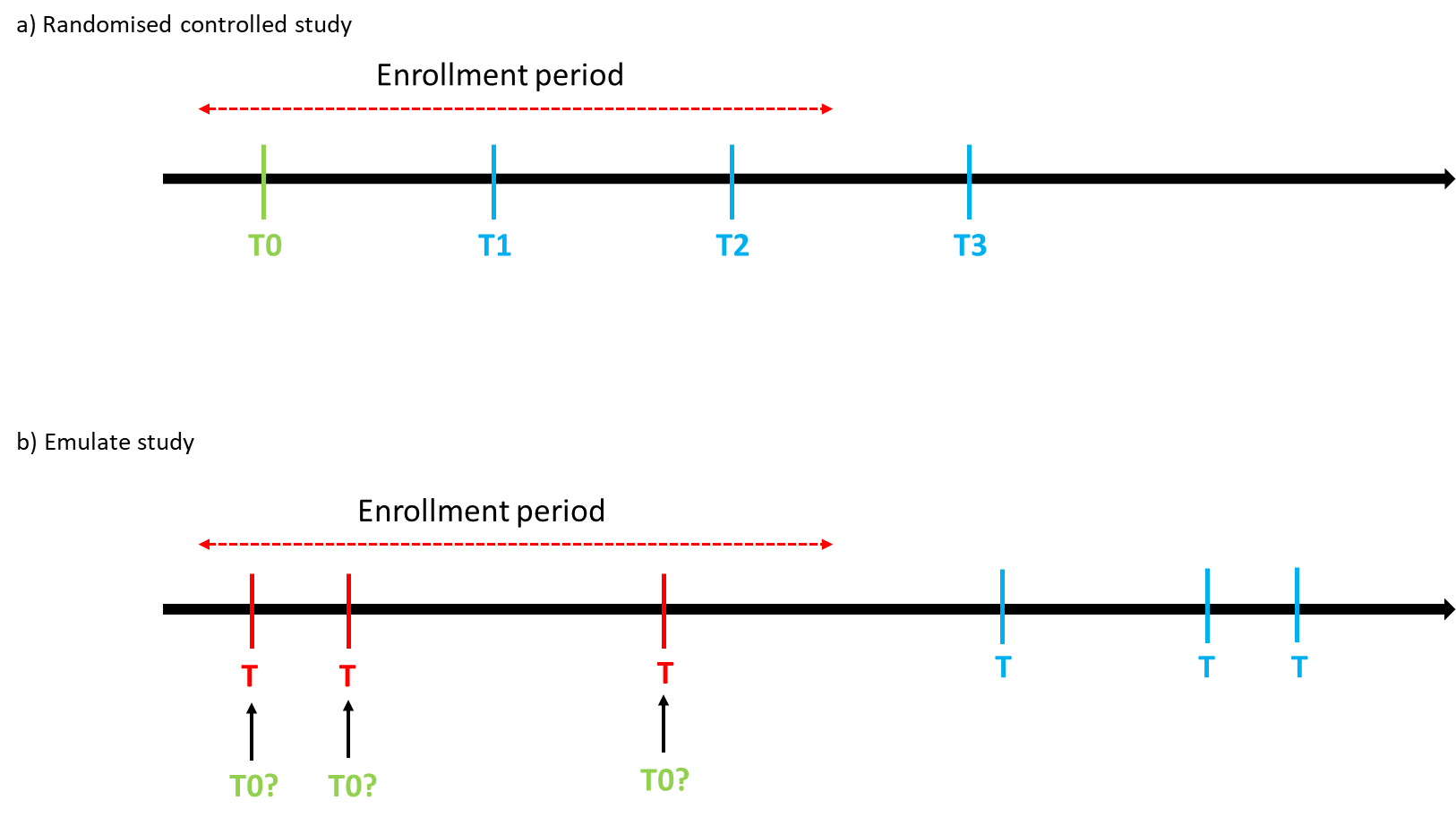
**

**eFigure 1**

**
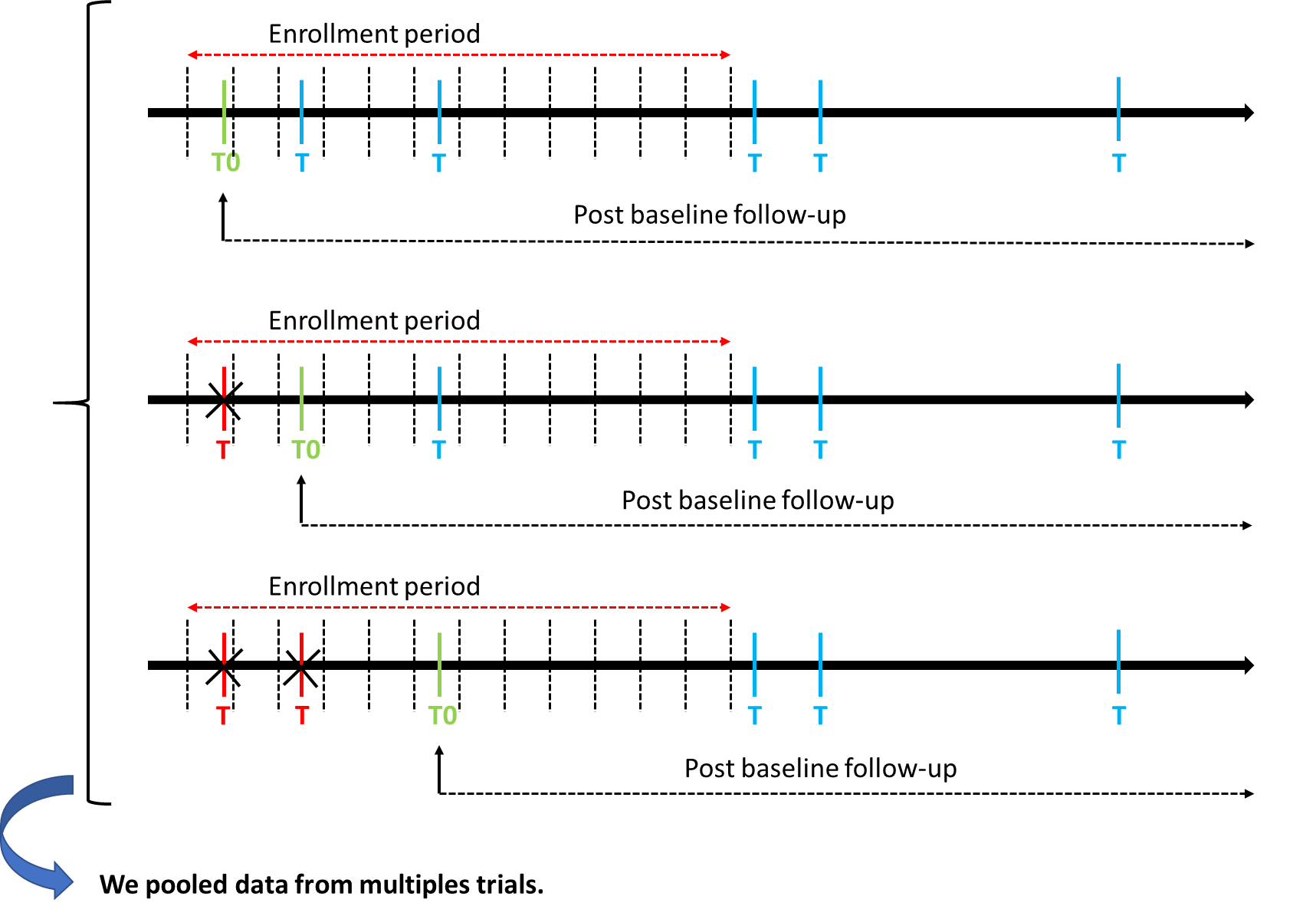
**

**eFigure 2**


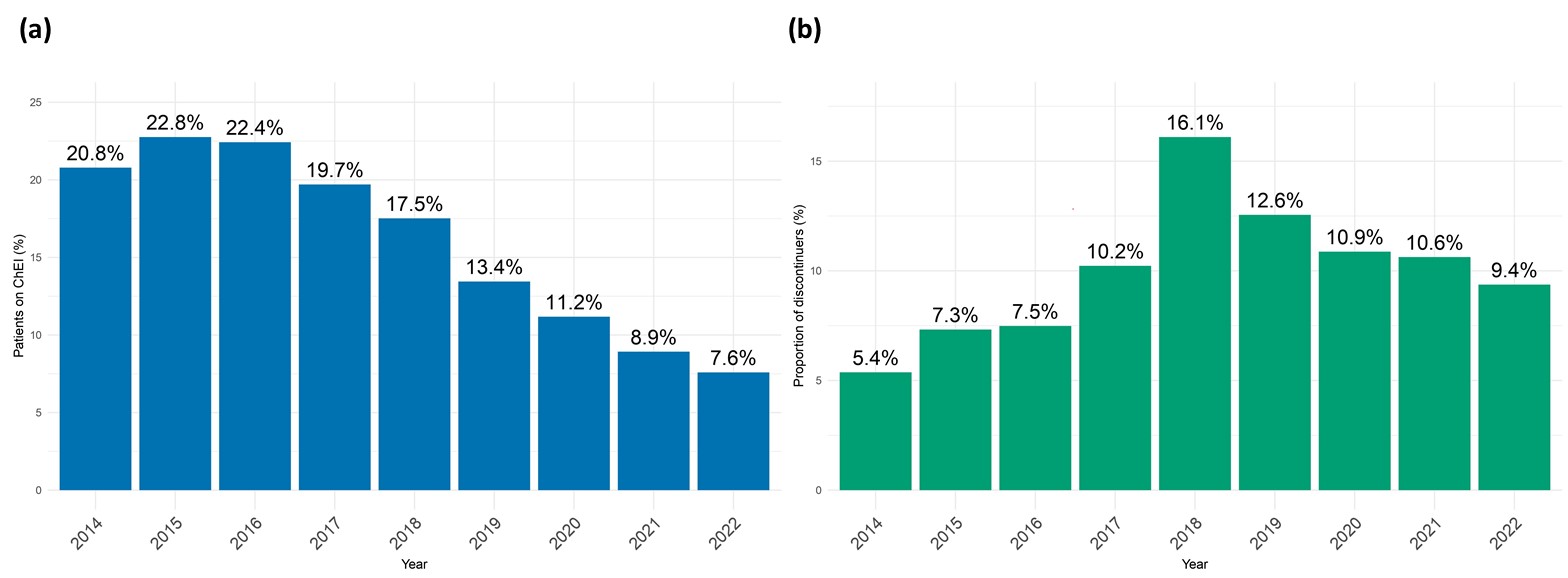


**eFigure 3**


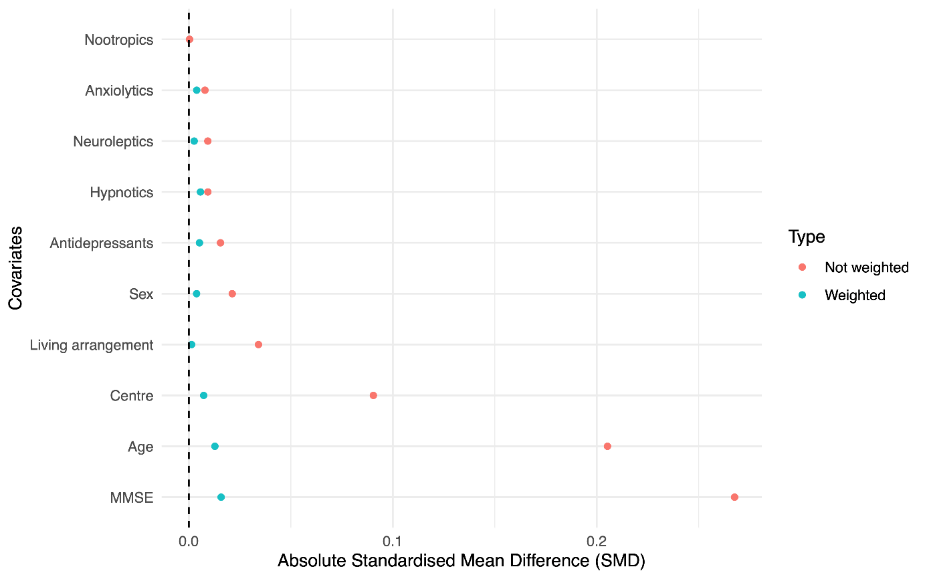


**eFigure 4**

**
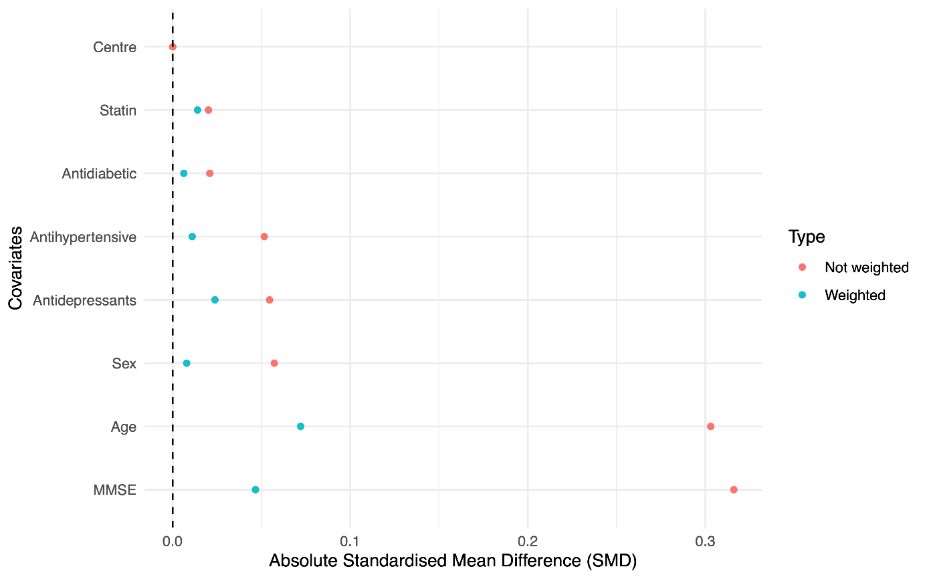
**

**eFigure 5**

**
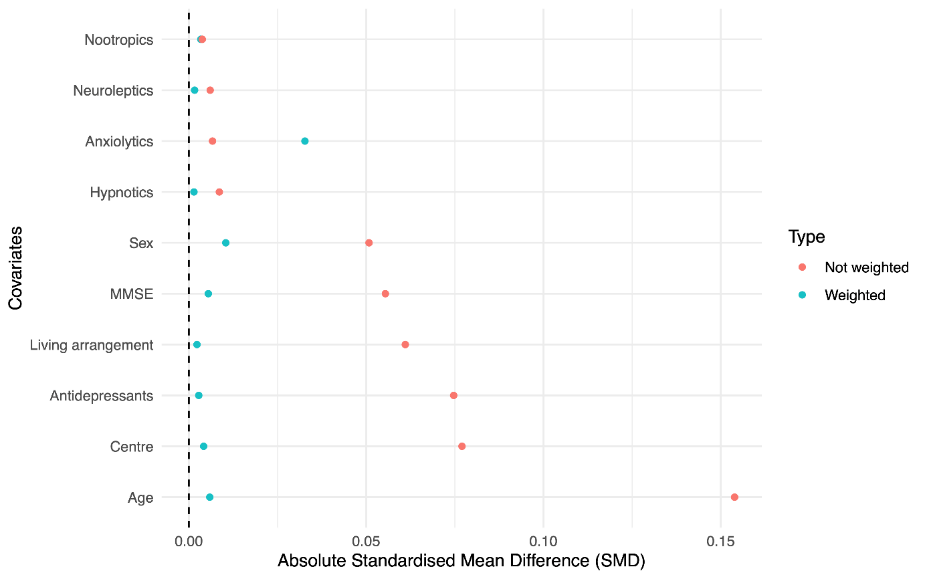
**

**eFigure 6**

**
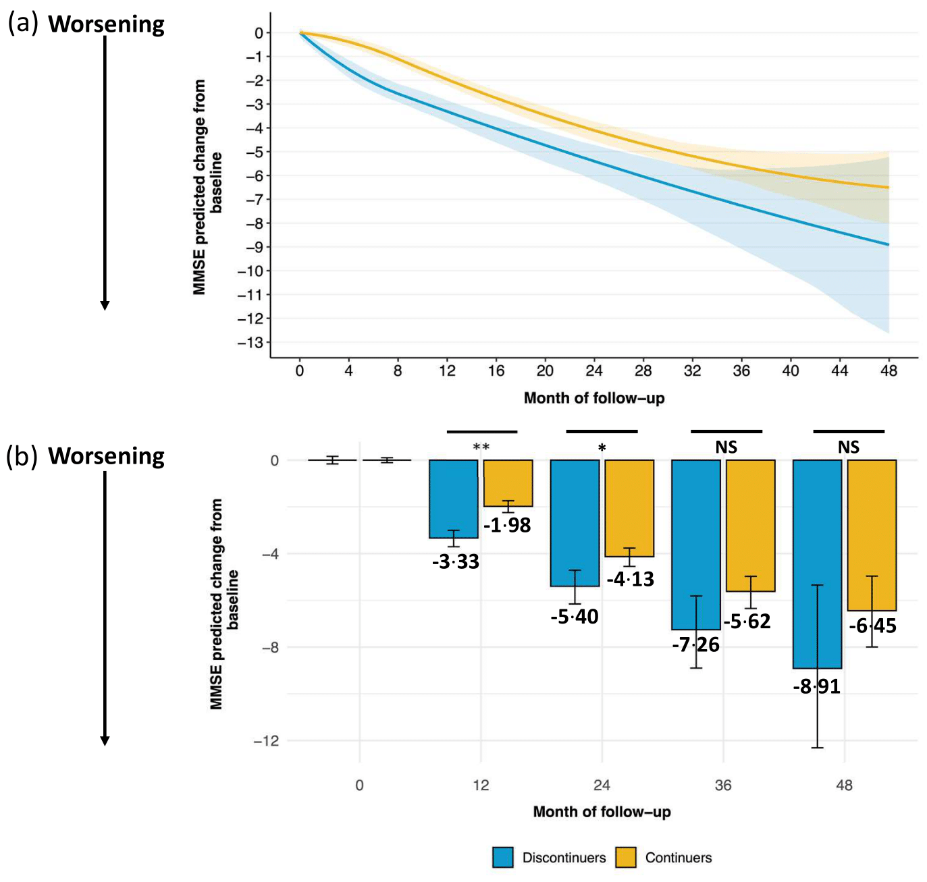
**

**eFigure 7**


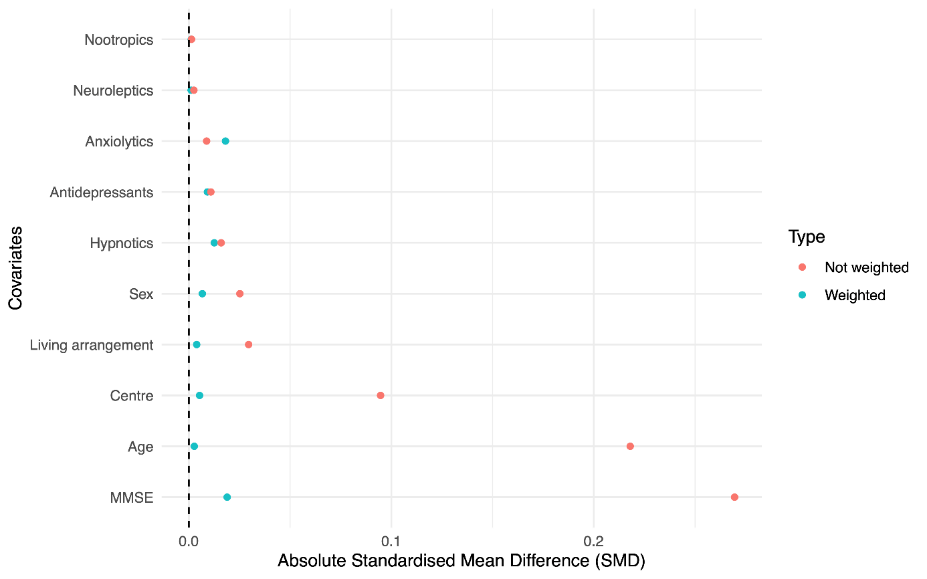


**eFigure 8**

**
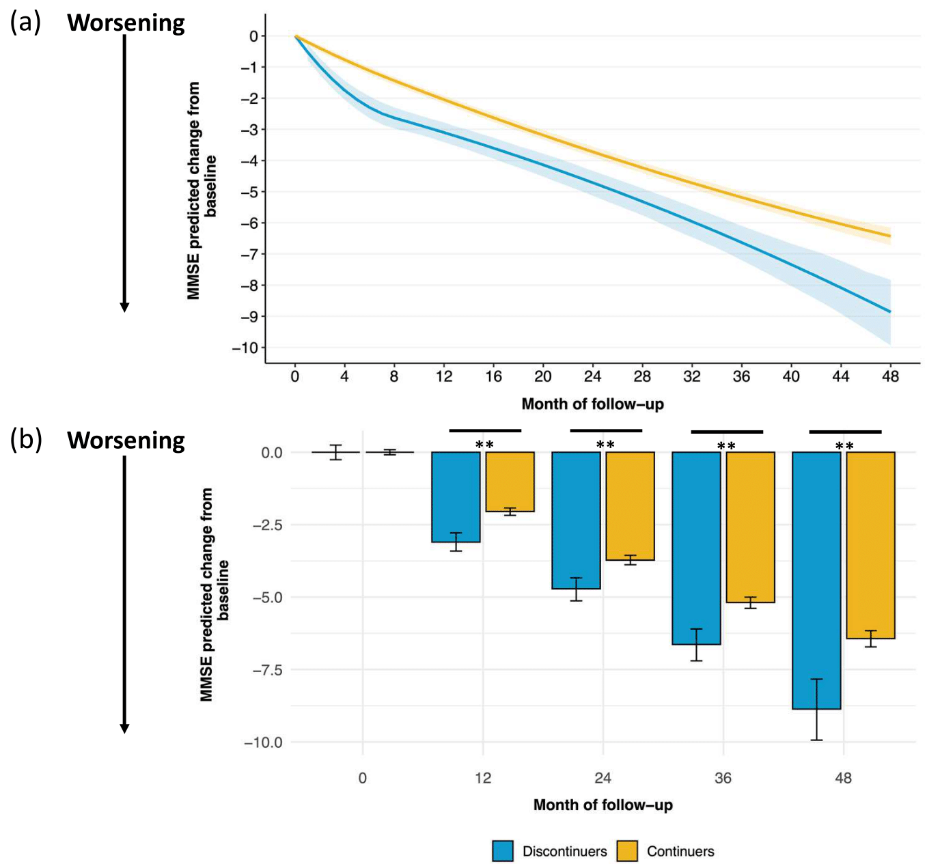
**

**eFigure 9**

**
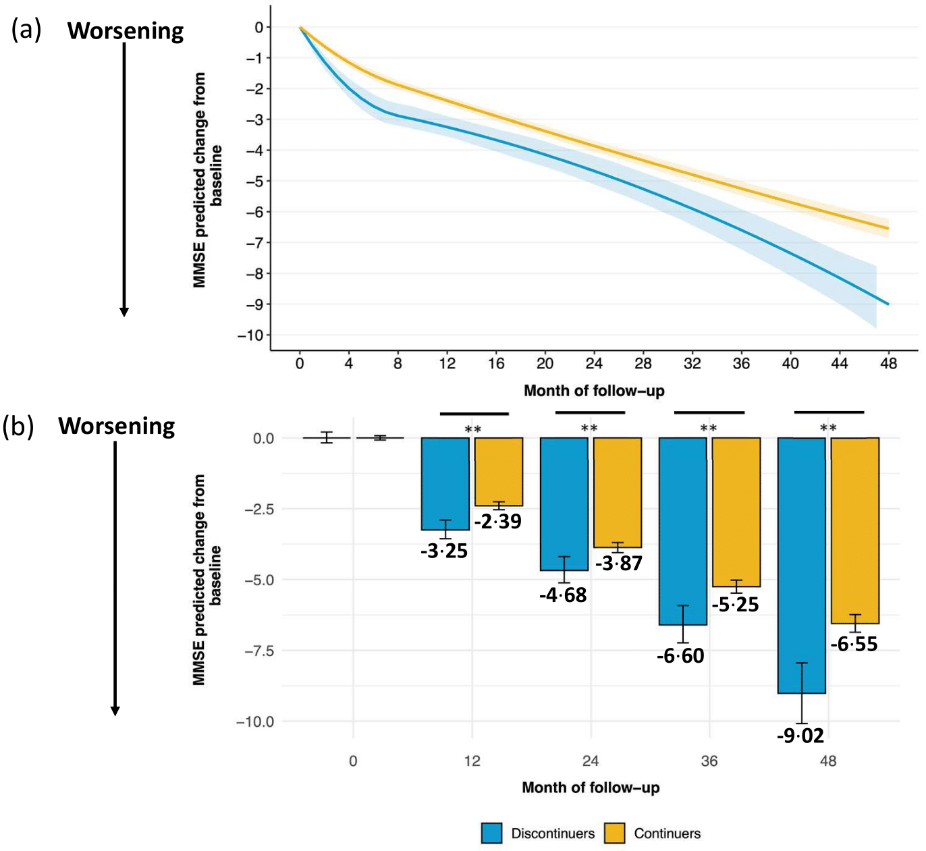
**

**eFigure 10**

.

**
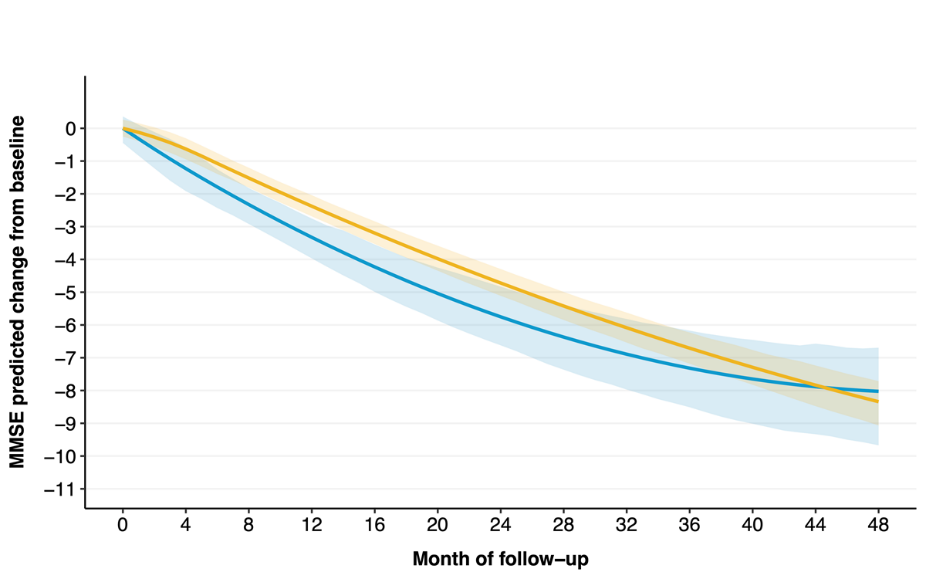
**

**eFigure 11**

**
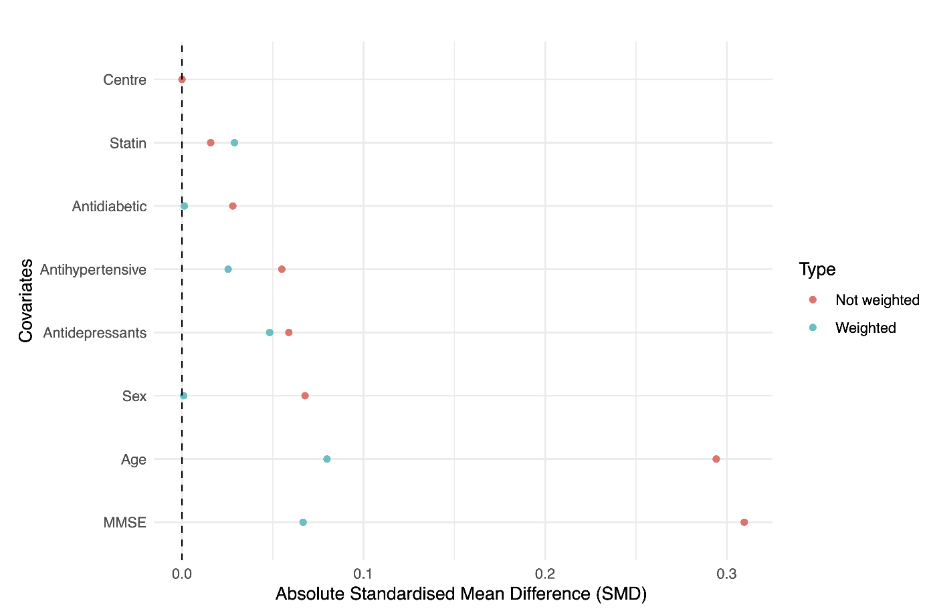
**

**eFigure 12**

**
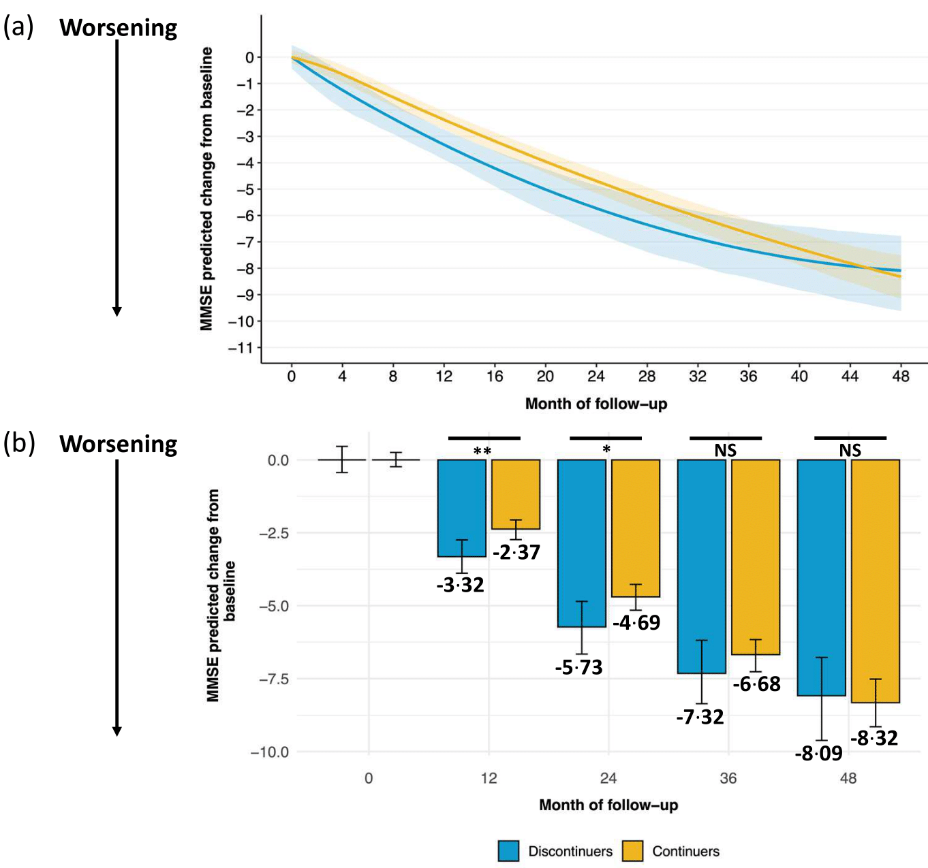
**

**eFigure 13**

**eTable 1**

|  | **Discontinuers (N=305)** | **Continuers (N=836)** | p-value |
| --- | --- | --- | --- |
| **MMSE at baseline, mean (SD)** | 10·6 (2·23) | 10·7 (2·25) | 0·44 |
| **Age at baseline (years), mean (SD)** | 81·1 (7·71) | 79.6 (8·47) | 0·005 |
| **Women, n (%)** | 239 (78·4%) | 592 (70·8%) | 0·014 |
| **Centre, n (%)** |  |  | 0·006 |
| Memory and research centre | 64 (21·0%) | 246 (29·4%) |  |
| Memory centre | 241 (79·0%) | 590 (70·6%) |  |
| **Education level, n (%)** |  |  | 0.76 |
| Primary | 164 (53·8%) | 460 (55·0%) |  |
| Secondary | 107 (35·1%) | 275 (32·9%) |  |
| Post-secondary | 34 (11·1%) | 101 (12·1%) |  |
| **Living arrangement, n (%)** |  |  | < 0·001 |
| Home | 261 (85·6%) | 773 (92·5%) |  |
| Nursing home | 44 (14·4%) | 63 (7·5%) |  |
| **Antidepressants, n (%)** | 140 (45·9%) | 326 (39·0%) | 0·042 |
| **Neuroleptics, n (%)** | 31 (10·2%) | 65 (7·8%) | 0·24 |
| **Anxiolytics, n (%)** | 49 (16·1%) | 144 (17·2%) | 0·71 |
| **Hypnotics, n (%)** | 13 (4·3%) | 33 (3·9%) | 0·95 |
| **Nootropics, n (%)** | 3 (1·0%) | 7 (0·8%) | 1 |

**eTable 2**

| **Shifted from the MAR** | **Treatment difference at one year**  **(disonctinuers vs. continuers)** | |
| --- | --- | --- |
|  | **Difference (95% CI)** | **p value** |
| 0 | 0·97 (0·68 to 1·27) | <0·001 |
| 0·5 | 0·91 (0·62 to 1·21) | <0·001 |
| 1 | 0·88 (0·58 to 1·18) | <0·001 |
| 1·5 | 0·83 (0·54 to 1·12) | <0·001 |
| 2 | 0·78 (0·46 to 1·10) | <0·001 |
| 2·5 | 0·73 (0·42 to 1·04) | <0·001 |
| 3 | 0·70 (0·40 to 1·00) | <0·001 |
| 3·5 | 0·63 (0·31 to 0·95) | <0·001 |
| 4 | 0·59 (0·29 to 0·90) | <0·001 |
| 4·5 | 0·53 (0·20 to 0·86) | <0·001 |
| 5 | 0.45 (0·15 to 0·76) | <0·001 |
| 5·5 | 0·39 (0·11 to 0·67) | <0·001 |
| 6 | 0·36 (0·06 to 0·65) | 0·002 |
| 6·5 | 0·30 (-0·01 to 0·61) | 0·06 |
